# Human-umbilical cord matrix mesenchymal cells improved left ventricular contractility independently of infarct size in swine myocardial infarction with reperfusion

**DOI:** 10.3389/fcvm.2023.1186574

**Published:** 2023-06-05

**Authors:** Luís Raposo, Rui J. Cerqueira, Sara Leite, Liliana Moreira-Costa, Tiago L. Laundos, Joana O. Miranda, Pedro Mendes-Ferreira, João Almeida Coelho, Rita N. Gomes, Perpétua Pinto-do-Ó, Diana S. Nascimento, André P. Lourenço, Nuno Cardim, Adelino Leite-Moreira

**Affiliations:** ^1^ Cardiology Department, Hospital de Santa Cruz - Centro Hospitalar de Lisboa Ocidental, Lisbon, Portugal; ^2^ Centro Cardiovascular, Hospital da Luz – Lisboa, Luz Saúde, Lisbon, Portugal; ^3^ Nova Medical School, Lisbon, Portugal; ^4^ Cardiovascular R&D Centre, UnIC@RISE, Department of Surgery and Physiology, Faculty of Medicine of the University of Porto, Porto, Portugal; ^5^ Department of Cardiothoracic Surgery, Hospital Universitário de São João, Porto, Portugal; ^6^ Anta Family Health Unit, Espinho/Gaia Healthcare Centre, Espinho, Portugal; ^7^ ICBAS- Instituto de Ciências Biomédicas Abel Salazar, Universidade do Porto, Porto, Portugal; ^8^ I3S – Instituto de Investigação e Inovação em Saúde, Universidade do Porto, Porto, Portugal; ^9^ INEB – Instituto Nacional de Engenharia Biomédica, Universidade do Porto, Porto, Portugal; ^10^ Paris-Porto Pulmonary Hypertension Collaborative Laboratory (3PH), UMR_S 999, INSERM, Université Paris-Saclay, Paris, France; ^11^ Department of Anesthesiology, Hospital Universitário de São João, Porto, Portugal

**Keywords:** umbilical-cord, mesenchymal cells, MSC, myocardial infarction, reperfusion

## Abstract

**Background:**

Human umbilical cord matrix-mesenchymal stromal cells (hUCM-MSC) have demonstrated beneficial effects in experimental acute myocardial infarction (AMI). Reperfusion injury hampers myocardial recovery in a clinical setting and its management is an unmet need. We investigated the efficacy of intracoronary (IC) delivery of xenogeneic hUCM-MSC as reperfusion-adjuvant therapy in a translational model of AMI in swine.

**Methods:**

In a placebo-controlled trial, pot-belied pigs were randomly assigned to a sham-control group (vehicle-injection; *n* = 8), AMI + vehicle (*n* = 12) or AMI + IC-injection (*n* = 11) of 5 × 10^5^ hUCM-MSC/Kg, within 30 min of reperfusion. AMI was created percutaneously by balloon occlusion of the mid-LAD. Left-ventricular function was blindly evaluated at 8-weeks by invasive pressure-volume loop analysis (primary endpoint). Mechanistic readouts included histology, strength-length relationship in skinned cardiomyocytes and gene expression analysis by RNA-sequencing.

**Results:**

As compared to vehicle, hUCM-MSC enhanced systolic function as shown by higher ejection fraction (65 ± 6% vs. 43 ± 4%; *p* = 0.0048), cardiac index (4.1 ± 0.4 vs. 3.1 ± 0.2 L/min/m^2^; *p* = 0.0378), preload recruitable stroke work (75 ± 13 vs. 36 ± 4 mmHg; *p* = 0.0256) and end-systolic elastance (2.8 ± 0.7 vs. 2.1 ± 0.4 mmHg*m^2^/ml; *p* = 0.0663). Infarct size was non-significantly lower in cell-treated animals (13.7 ± 2.2% vs. 15.9 ± 2.7%; Δ = −2.2%; *p* = 0.23), as was interstitial fibrosis and cardiomyocyte hypertrophy in the remote myocardium. Sarcomere active tension improved, and genes related to extracellular matrix remodelling (including MMP9, TIMP1 and PAI1), collagen fibril organization and glycosaminoglycan biosynthesis were downregulated in animals treated with hUCM-MSC.

**Conclusion:**

Intracoronary transfer of xenogeneic hUCM-MSC shortly after reperfusion improved left-ventricular systolic function, which could not be explained by the observed extent of infarct size reduction alone. Combined contributions of favourable modification of myocardial interstitial fibrosis, matrix remodelling and enhanced cardiomyocyte contractility in the remote myocardium may provide mechanistic insight for the biological effect.

## Introduction

Cell therapy has been extensively investigated as a potential treatment to improve left ventricle (LV) function after acute myocardial infarction (AMI). Different cell types have entered the experimental arena, but most studies have used bone marrow-derived cells (BMCs) (1). Unfortunately, the impressive biological effects observed in small animal models have not been consistently reproduced in clinical trials. Despite enhancement of LV ejection fraction, as well as limitation of scar formation and chamber remodeling have been demonstrated, the impact on clinical outcomes has been perceived as discouraging (2, 3). Impaired functional potency of autologous cells, which are obtained from patients with comorbidities, has been considered a limitation for clinical translation that may be overcome by allogeneic cells (4–6). Although most of research with allogeneic cell products has focused on adult mesenchymal stromal cells (MSC) (7), the higher potency of fetal MSCs may provide enhanced biological effects (8). The umbilical cord is an abundant source of fetal MSC, which can be derived from the cord blood, perivascular tissue or the connective tissue matrix (Wharton's Jelly) (9). Prior studies from our group in a rodent permanent left-anterior descending artery (LAD)-ligation AMI model have shown that acute intra-myocardial delivery of human-umbilical cord matrix-derived MSC (hUCM-MSC) sustainedly attenuates remodeling by proangiogenic, antiapoptotic and endogenous cell-activation mechanisms (10, 11). However, studies testing hUCM-MSC in larger animal models of AMI with reperfusion are lacking (12). Such preclinical models would better mimic the typical setting of clinical AMI, in which reperfusion is achieved in most cases. Also, mitigation of reperfusion injury is still clinically unmet (13). Thus, we designed a study to test the safety (concerning potential cell-related myocardial perfusion impairment) and efficacy of intra-coronary administration of a clinical grade GMP-compliant hUCM-MSC product, in a large-animal preclinical model of AMI with reperfusion.

## Materials and methods

### Study design

**Figure 1:**
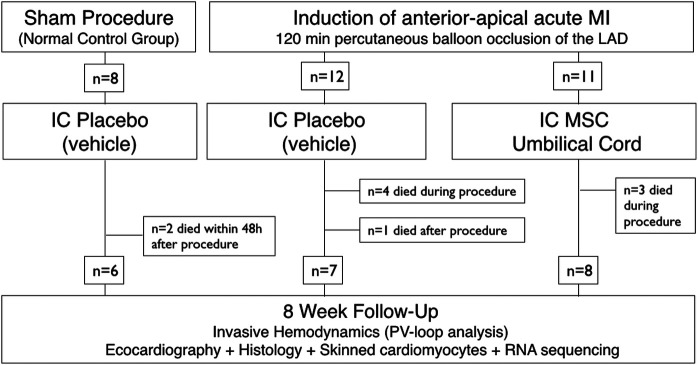
Study flowchart.

Adult (1-year old) male Vietnamese pot-bellied pigs were block-randomized in blocks of 8 (with a 2:3:3 allocation ratio) to sham-vehicle (*n* = 8), AMI-vehicle (*n* = 12) and AMI-intracoronary injection of hUCM-MSC (*n* = 11) (Figure 1). Randomization order was electronically generated and animals entered the study sequentially. hUCM-MSC or an equal volume of vehicle [phosphate buffered saline (PBS)] were infused within 30 min of reperfusion. Researchers other than the investigator responsible for preparing the cell product and assessing cell viability were blinded to allocation.

### Animal model

Anesthesia was induced, after a 12 h fast without water restriction, by intramuscular injection of ketamine (15 mg/Kg) and midazolam (0.5 mg/Kg). Upon peripheral venous catheterization in the ear, anesthesia was maintained by propofol (200 mg/Kg bolus and 100–200 mg/Kg/min infusion) with fentanyl (20 µg/Kg bolus and 3 mg/Kg/min infusion) as analgesic. Upon orotracheal intubation animals were mechanically ventilated with 100% oxygen, tidal volume set at 10 ml/Kg, respiratory rate at 20/min and positive end-expiratory pressure held at 5 cmH_2_O. Vascular sheaths (6-Fr) were placed percutaneously in a femoral artery and vein. Body temperature, EKG, oxygen saturation and hemodynamics were monitored throughout the procedure. Prior to induction of AMI anticoagulation was achieved with intravenous unfractionated heparin (200–250 IU/Kg), and intravenous amiodarone (75 mg over 10 min and 1 mg/Kg/h infusion) was administered to reduce ventricular arrhythmia. Cardioversion (360J monopolar) was undertaken according to standard indications. Additional boluses of lidocaine (1–3 mg/kg) were given in case of cardioversion-refractory or persistent ventricular tachycardia. Epinephrine and atropine were used whenever needed, as indicated (14). The left main coronary artery was selectively engaged with a 6-Fr JL-3 guiding catheter (Cordis®, Miami Lakes, FL), the left anterior descending artery (LAD) was located angiographically, and a 0.014' guide-wire was placed in the distal vessel. To create an anterior-apical infarction, a 2.5–3 mm angioplasty balloon-catheter (sized visually to the reference vessel diameter) was advanced into the LAD and inflated distally to the largest diagonal branch for 120 min. Reperfusion was achieved by balloon deflation.

Animals were housed according to standard recommendations and their behavior, excreta and feeding pattern were closely monitored by veterinarian staff (15). Throughout follow-up, animals were given a beta-blocker (atenolol 25 mg daily) and an angiotensin-converting enzyme inhibitor (ramipril 2.5 mg daily), starting on the second day after AMI (16). Despite the xenogeneic nature of the cell product, no immune suppression was used.

### Characteristics of the hUCM-MSC cell product

Human umbilical cord matrix-derived mesenchymal cells were obtained and prepared using certifiable advanced therapy medicinal product (ATMP)-compliant protocols and a patented proprietary technology (PCT/IB2008/054067; WO 2009044379), as described elsewhere (17). Biological characterization of the cell product and release criteria have been previously validated (7, 17) and are summarized in the Supplementary Material.

### Preparation and delivery of hUCM-MSC

The cryopreserved cell product was thawed and reconstituted to obtain a final concentration of 0.5 × 10^6^ cells/ml, according to manufacturer's specifications. In each batch, cell viability was assessed by the trypan blue assay (Supplementary Figures S1B). Upon stable rhythm and hemodynamics and up to 30 min after reperfusion, animals underwent intra-coronary infusion of 0.5 million hUCM-MSC per Kilogram of body weight or an equal volume of placebo (PBS-vehicle solution). Intracoronary injections were performed using a tapered 1.8/2.6-Fr coronary microcatheter (Finecross MG®; Terumo® Interventional Systems, Summerset, NJ) at a constant rate of 1 ml/min (0.5 × 10^6^ hUCM-MSC/min), with permanent low-grade agitation of the solution to maintain cell suspension. In all groups, coronary flow was assessed by continuous of Doppler-derived average peak velocity (APV in cm/s) using a Doppler-tipped guidewire (ComboWire XT®; Volcano Corporation®, San Diego, CA), which measures instantaneous coronary flow velocity throughout the cardiac cycle.

### Invasive hemodynamics

Eight-weeks after AMI, upon anesthesia induction and mechanical ventilation as previously described, closed-chest LV pressure-volume (PV) recordings were performed with a 5-Fr pigtail multi-segment pressure-volume conductance catheter (Ventri-Cath™ 507; Millar Instruments, Inc., Houston, TX) inserted through the femoral artery and guided to the LV apex with a 7-Fr 90 cm sheath (Cordis®, Miami Lakes, FL). Evaluation of load independent parameters was performed by transient occlusion of the inferior vena cava with a 25 mm-diameter balloon catheter (NuCLEUS™, NuMED™, Hopkinton, NY) with ventilation suspended at end-expiration. Cardiac output was measured continuously by thermodilution using a Swan-Ganz catheter (Edwards LifeSciences™, CL) inserted through the right internal jugular vein (8-Fr). Data was continuously acquired and recorded with the PowerLab 16/35 16-channel acquisition system, and analyzed offline using the LabChart 7 Pro software (ADInstruments, Oxford, UK). Volume signal was calibrated for parallel conductance by 10 ml 10% saline injection and for slope factor α by simultaneous measurement of cardiac output. Volume data was calibrated for swine body surface area according to corrections for mini-pig strains (18). End-systolic and end-diastolic PV relationships were obtained by linear fitting and exponential fitting with a pressure asymptote, respectively.

### Transthoracic echocardiogram

Imaging was undertaken for morphological confirmation of segmental changes after induction of myocardial infarction at baseline and before PV terminal evaluations. Obtained parameters were not meant for endpoint evaluation (Supplementary Table S3 and Supplementary Figure S5). Transthoracic echocardiograms were performed using an ACUSON™ Sequoia C152 System (Siemens Medical™) equipped with a 3 MHz phased-array probe (3V2c, Siemens Medical™).

### Biological samples

A 20 ml blood sample was collected for biochemical analysis (namely of serum NTproBNP and galectine-3) at the beginning of terminal evaluations, before any further invasive procedures. Upon completion of experimental evaluation, a median thoracotomy was performed under deep anesthesia and animals were euthanized by exsanguination. Beating hearts were harvested and perfused anterogradely with cold crystalloid hyperkalemic solution. Tissue biopsies were collected from the infarct zone, border zone and the remote LV myocardium. The remaining heart was processed for paraffin embedding.

### Histology

For analysis of infarct size, whole hearts were protected from frost bite in clear wrap and incubated at −20°C for 2 h before sectioning with a meat slicer at 50 mm intervals from LV apex to base. Sections were incubated in 1% 2,3,5-Triphenyltetrazolium chloride (TTC) at 37°C for 7 min with viable tissue showing red coloring, fixed in 10% formalin for ∼10 min, digitized and subjected to semi-automatic morphometric analysis. Infarcted area, infarcted midline length and LV chamber area were calculated using the semi-automatic MIQuant Software adapted for TTC staining (19). Collagen deposition analysis of midline scar length, the circumference of scarred LV midway between the epicardial and endocardial surfaces and LV infarcted wall thickness were determined manually using ImageJ. For each heart, results represent the average of all infarcted sections. For microscopy studies, nuclei were pre-stained with Celestine Blue solution after staining with Gill's Hematoxylin and incubation for 1 h in aqueous Bouin solution to promote uniform staining and then stained with H&E and Masson's Trichrome (Sigma-Aldrich).

### Skinned cardiomyocytes

The detailed protocol for specimen preparation has been described elsewhere (20). In brief, remote myocardial tissue was cut into small pieces, mechanically disrupted and incubated for 5 min in relaxing solution supplemented with 0.5% Triton X-100 in order to remove membrane structures. After washing, single cardiomyocytes were attached with glue between a force transducer and an electromagnetic motor. Passive tension- and active tension-sarcomere length (SL) relationships ranging between 1.8 and 2.3 μm were acquired at 15°C with 0.1 μm step increases. Force measurements were normalized to cardiomyocyte cross sectional area.

### RNA sequencing

A total of 400 ng of extracted RNA from the remote LV myocardium was used for RNA sequencing after ribosomal and mitochondrial RNA depletion. Ion Torrent sequencing libraries were prepared, and sequencing was performed in an Ion S5XL™ sequencer (Thermo-Fisher). On average, about 15 million reads were obtained for each sample. Sample read quality was determined using FASTQC, low quality sequences were dropped (Phred score <30) and Trimmomatic was used to remove adapters (21). Resulting reads were aligned to the reference pig genome (Sus_scrofa.Sscrofa11.1.92) with HISAT2 (22). FeatureCounts was used to obtain gene counts in Galaxy online software (23). Differentially expressed gene (DEG) analysis was performed using edgeR in Galaxy (ver.3.36.0), using DEG selection criteria of [log2(Fold Change)] >1 or <−1 and false-discovery rate (FDR) <0.05. Gene ontology analysis was performed using Enrichr (GO Biological Process 2021) (24, 25). Principal coordinate analysis (PCA) coordinates were obtained in Galaxy using ggplot2 (ver. 3.3.5). Expression heatmaps were assembled using Heatmapper online software (26), using complete clustering and Pearson distance measuring method.

### Statistical analysis

Sample size was determined to achieve a power of 80% with a alpha level (type I error) of 0.05 for a 2-sided comparison of means in one-way ANOVA to detect a 20% increase in P-V loop derived ejection fraction in the active treatment group compared with vehicle (PASS 15 NCSS), based on previous data for ejection fraction after AMI in similar animal models (40% ejection fraction in AMI-placebo and a standard deviation of 8% for groups) (16, 27).

Shapiro-Wilk's test was used to assess normality of residuals and the Levene test to probe equality of variances. Datasets following a Gaussian distribution and showing same standard deviation were analyzed using one-way ANOVA, followed by the Tukey's or the Fisher's Least Significant Difference *post-hoc* tests for multiple comparisons, as appropriate. Non-parametric data was tested with Kruskal-Wallis test, followed by the FDR method of Benjamini and Hochberg adjustment for multiple comparisons. End-systolic and end-diastolic pressure volume relationships were compared by analysis of covariance with end-systolic elastance and chamber stiffness constant (β) as dependent variables and other coefficients as covariates.

Statistical significance was set at a 2-sided *p*-value of 0.05.

IBM™ SPSS Statistics™ Version 23 and Prism Version 9.3.1 (GraphPad Software, LLC.) were used for statistical analysis and graphical data representation.

## Results

Thirty-one animals weighing 33.1 ± 7.4 kg were used in this experiment (Supplementary Table S1). Seven animals died during AMI-induction (mostly due to fatal arrythmias), yielding an AMI-related mortality rate of 30%. Another 3 animals died after the procedure, 2 of which in the sham group, within 48-h of instrumentation (Figure 1). Twenty-one animals completed follow-up and were available for outcome evaluation (6, 7 and 8 in groups sham-vehicle, AMI-vehicle and AMI-hUCM-MSC, respectively).

### Coronary blood flow during hUCM-MSC delivery

**Figure 2:**
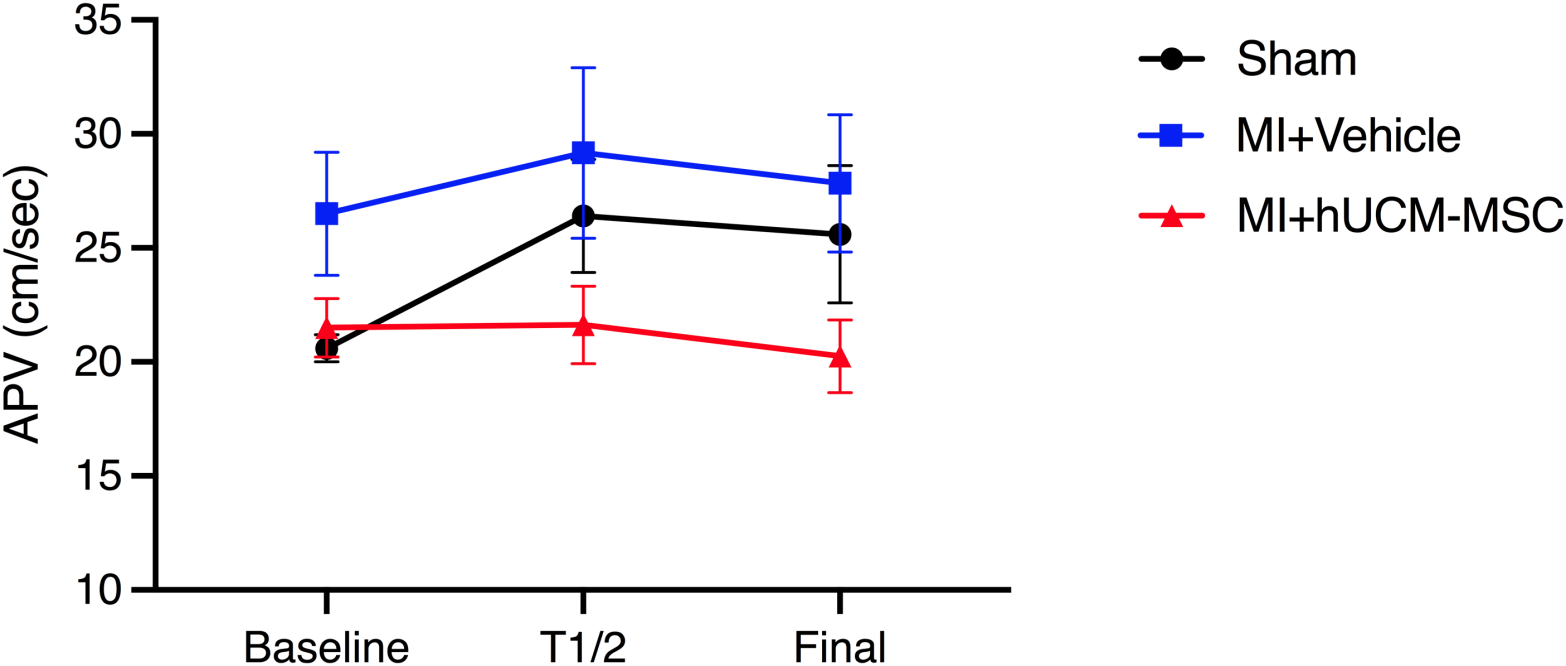
Doppler-derived coronary flow velocity. At the end of the injection, APV was higher in vehicle injected animals (with and without MI), as compared to hUCM-MSC (26.8 ± 6.8 vs. 20.5 ± 4.5; ANOVA *p* = 0.03). In infarcted animals injected with hUCM-MSC, final APV did not decrease significantly as compared do baseline (20.3 ± 4.5 vs. 21.5 ± 3.6; Related samples Wilcoxon Signed rank test *p *= 0.25). Symbols represent geometric means and bars represent standard deviation. APV, average peak velocity; T1/2—half time injection.

Appropriate doppler signals were obtained in 19 animals. Baseline average peak velocity (APV) trended higher in infarcted animals as compared to sham controls (23.6 ± 5.5 cm/s vs. 20.6 ± 1.3 cm/s; *p* = 0.074). During injection, flow velocity increased in vehicle, but not in hUCM-MSC (21.5 ± 3.6 to 20.3 ± 4.5 cm/s from baseline to terminus, respectively; *p* = 0.24). Results of real-time intracoronary doppler assessment are summarized in Figure 2.

### hUCM-MSC improved systolic function

**Figure 3:**
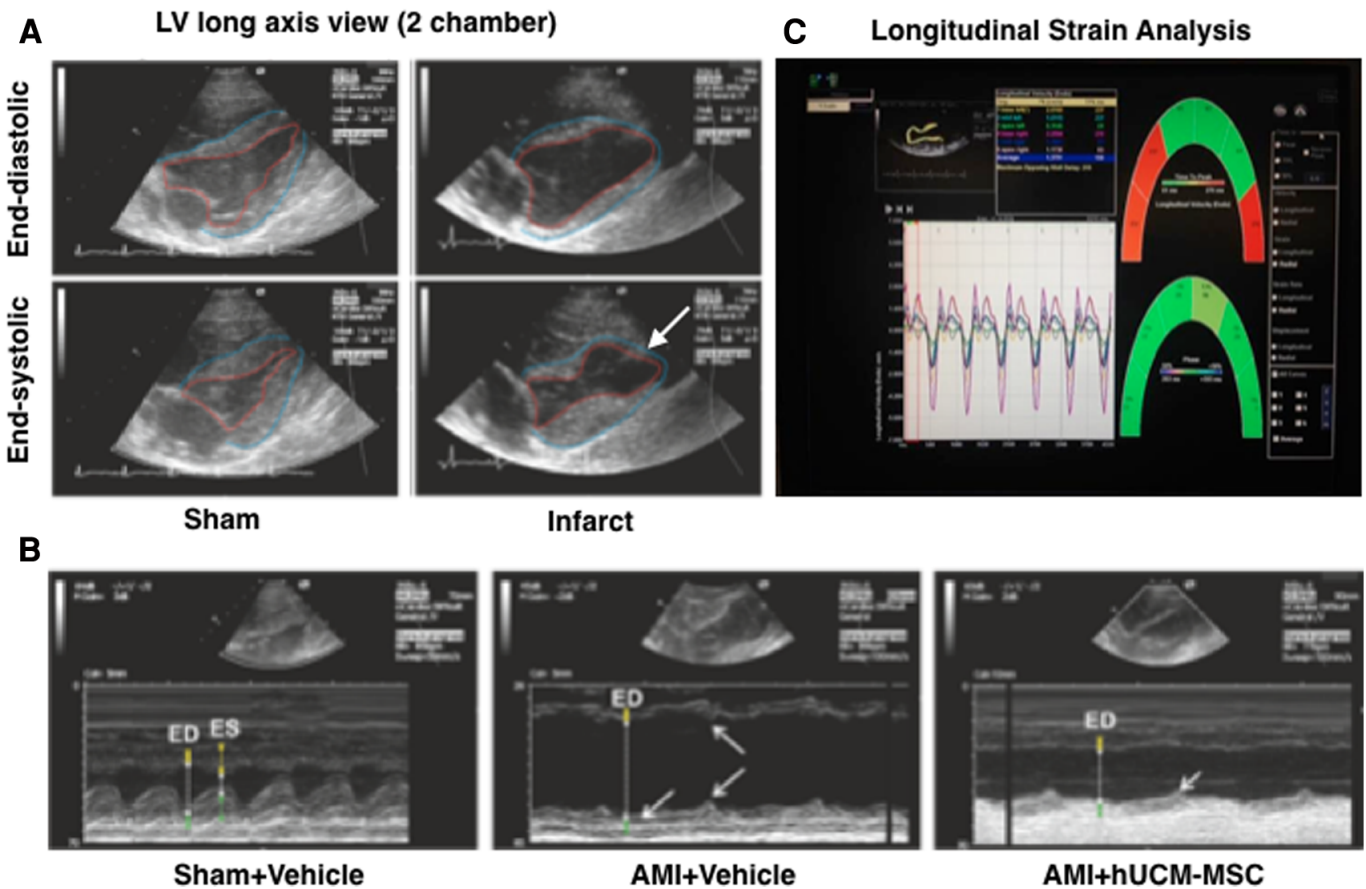
Representative images of transthoracic echocardiogram. Bidimensional images (**A**) in infarcted animals show apical akinesia (white arrow) and reduced wall thickness. In M-mode (**B**) measurements of Interventricular septum thickness (yellow), left ventricular cavity dimension (white) and left ventricular lateral wall thickness (green) are indicated at end-diastole and end-systole (ED and ES, respectively). Notice the dilation in AMI pigs, marked wall dyskinesia (indicated by arrows) and reduced systolic wall thickening. An example of speckle tracking longitudinal strain analysis is shown (**C**).

Animals that underwent AMI showed acute apical akinesia/dyskinesia and systolic dysfunction. Representative examples of echocardiograms obtained after AMI-induction are shown in Figure 3.

**Figure 4:**
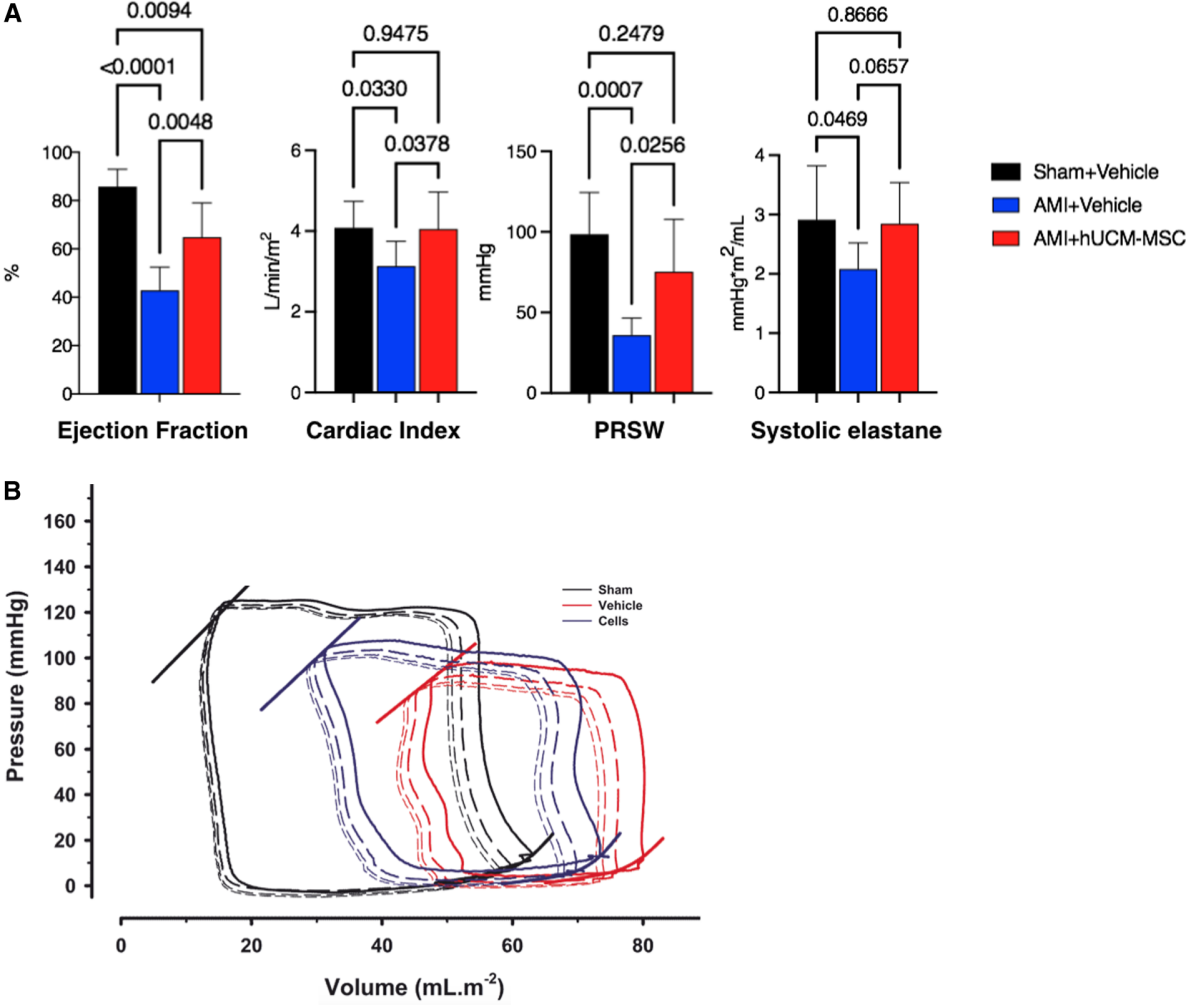
(**A**) Invasive hemodynamic parameters and (**B**) representative pressure-volume loops of vehicle-injected sham (sham + vehicle), vehicle-injected myocardial infarction (AMI + vehicle), and umbilical cord stromal cell-injected myocardial infarction pigs (AMI + hUCM-MSC). End-systolic and end-diastolic pressure-volume relationships are plotted on the left upper and right lower corner of the loops, respectively. Bars represent mean and standard deviation. *p*-values are described for all comparisons between groups. PRSW, preload recruitable stroke work.

Good quality PV data could not be obtained in 2 animals from AMI-hUCM-MSC due to procedure related complications (manipulation-related cardiac tamponade, not related to cell therapy). Results of invasive hemodynamic evaluation are summarized in Figure 4 and detailed on Supplementary Table S2.

At 8-weeks post-instrumentation, infarcted pigs showed cardiac dilation and impaired contractility compared with sham pigs. In both AMI groups EDVi was larger, but less so in animals treated with intra-coronary hUCM-MSC, as compared to vehicle (73 ± 8 vs. 80 ± 5 ml/m^2^, respectively; *p* = 0.115). Compared to their vehicle-injected counterparts, AMI-hUCM-MSC animals showed significant improvements in indexed end-systolic volume (ESVi) (28.5 ± 12.5% vs. 47.4 ± 8.4 ml/m^2^; *p *= 0.004), ejection fraction (EF) (65 ± 6% vs. 43 ± 4%; *p *= 0.0048) and cardiac index (CI) (4.1 ± 0.4 vs. 3.1 ± 0.2 L/min/m^2^; *p *= 0.0378). Intrinsic myocardial contractility was also improved by hUCM-MSC as assessed by preload recruitable stroke work (PRSW) (75 ± 13 vs. 36 ± 4 mmHg; *p *= 0.0256) and end-systolic elastance (Es*_i_*) (2.8 ± 0.4 vs. 2.1 ± 0.7 mmHg*m^2^/ml; *p *= 0.0657). Differences between groups for relaxation (*t*), end-diastolic pressure or end-diastolic compliance, as appraised by chamber stiffness constant *b*, were smaller and not statistically significant, although a clear trend for a leftward-shift of the end-diastolic PV relationship was obvious in both AMI-groups. Importantly, systemic vascular resistances (SVRi) were significantly lower in infarcted animals treated with hUCM-MSC as compared to vehicle [18.6 ± 6.24 vs. 26.3 ± 3.1 mmHg/(L/min/m^2^); *p* = 0.047] and comparable to sham controls [20.2 ± 5.7 mmHg/(L/min/m^2^); *p* = 0.592].

### hUCM-MSC decreased fibrosis and cardiomyocyte hypertrophy

**Figure 5:**
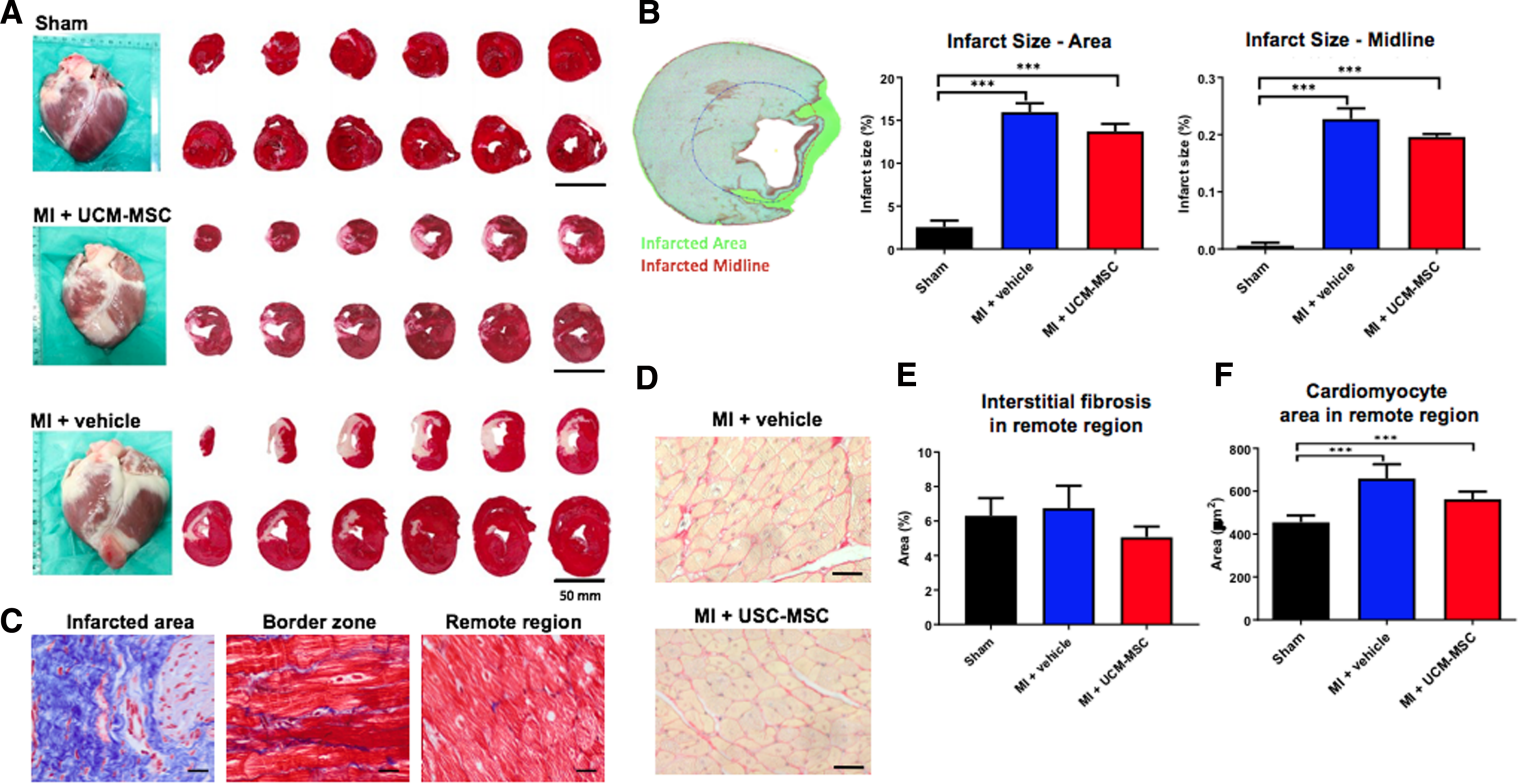
Semi-automated quantification of infarct size. Triphenyl tetrazolium chloride (TTC) staining (**A,B**) and Quantification of interstitial fibrosis and cardiomyocyte hypertrophy in the remote myocardium (Panels **C–F**). In Panels **B,E,F**, bars represent the average (and standard error) of all hearts in each group. Symbols (***) denote significant differences between groups (*p* < 0.05). Differences favoring AMI-hUC-MSC vs. AMI-vehicle were not statistically significant.

Despite the conspicuous trend, infarct size reduction was not statistically significant in AMI-hUCM-MSC as compared with AMI-vehicle (Figure 5A,B), assessed either as percentage of LV area (13.7 ± 2.2% vs. 15.9 ± 2.7%, *p *= 0.23) or as midline scar length (19.6 ± 1.3 vs. 22.7 ± 4.9; *p *= 0.16). An exploratory analysis showed an inverse correlation between cell viability in the active treatment group and final histological infarct size (Supplementary Figure S2). Based on this observation, a non-prespecified *post hoc* comparison of scar extent was performed after excluding the one animal from the hUC-MSC group with a viable cell count bellow the group's standard deviation (<80%). This analysis evidenced a greater difference in fibrotic infarct size favoring hUCM-MSC treatment vs. vehicle, which was statistically significant (Δ = −2.9%; ANOVA *p *= 0.043; Supplementary Figure S3). Both AMI-groups showed heavy deposition of thick collagen bundles in the ischemic area with extension to the interstitial space of the border zone (Figure 5C). Quantification of collagen fibers and of cardiomyocyte cross-sectional area of Pico-Sirius Red stained sections further demonstrated that hearts from hUCM-MSC-treated animals exhibited less interstitial fibrosis and cardiomyocyte hypertrophy in the LV remote zone compared do AMI-vehicle, although differences were not statistically significant (Figures 5D–F).

### Active and passive tension in skinned cardiomyocytes

**Figure 6:**
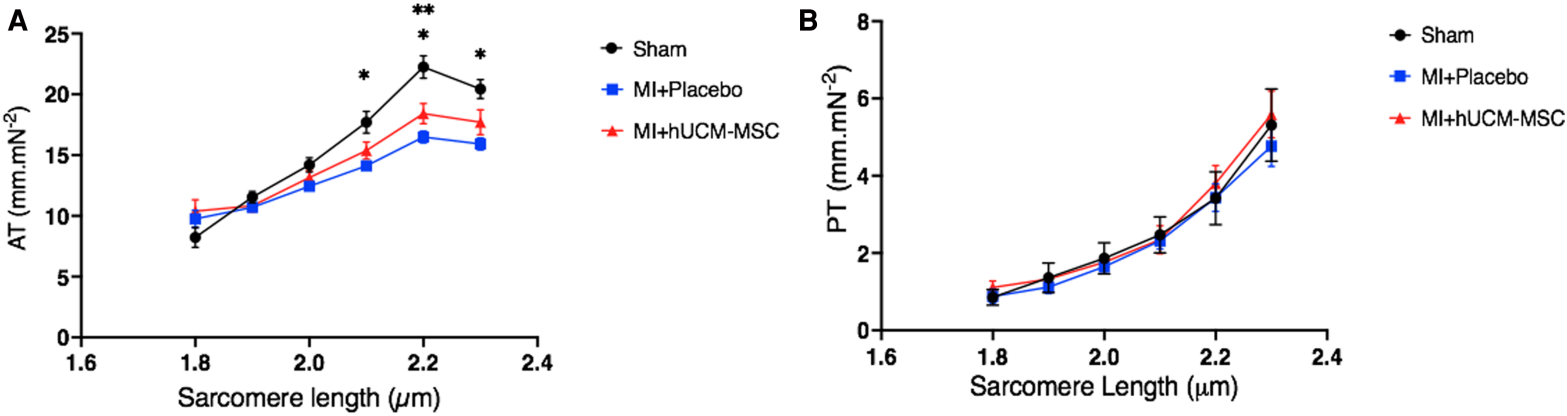
Active (**A**) and passive (**B**) length-tension relationship in isolated skinned cardiomyocytes from the remote myocardium. AT, active tension; PT, passive tension. *p < 0.05 for the comparison of sham vs. placebo: **p < 0.05 for the comparison of sham vs. cells. Regarding the comparisons of passive tension between groups (Panel **B**), *p*-values ranged between 0.57 and 0.99.

Active tension was lower in both MI groups, with sarcomeres from sham animals exhibiting higher active tension at most sarcomere lengths (SL). Also, the slope of the active length-tension relationship curve was lower in both MI groups. As compared to placebo, for increasing sarcomere length (>2 µm), the force of isometric contraction (active tension) was higher in cell-treated animals, and closest to active tension developed by sham-controls (Figure 6A). Passive length-tension relationship was similar across groups (Figure 6B).

### RNA sequencing unveils molecular alterations towards less ECM remodeling in UCM-MSC treated animals

**Figure 7:**
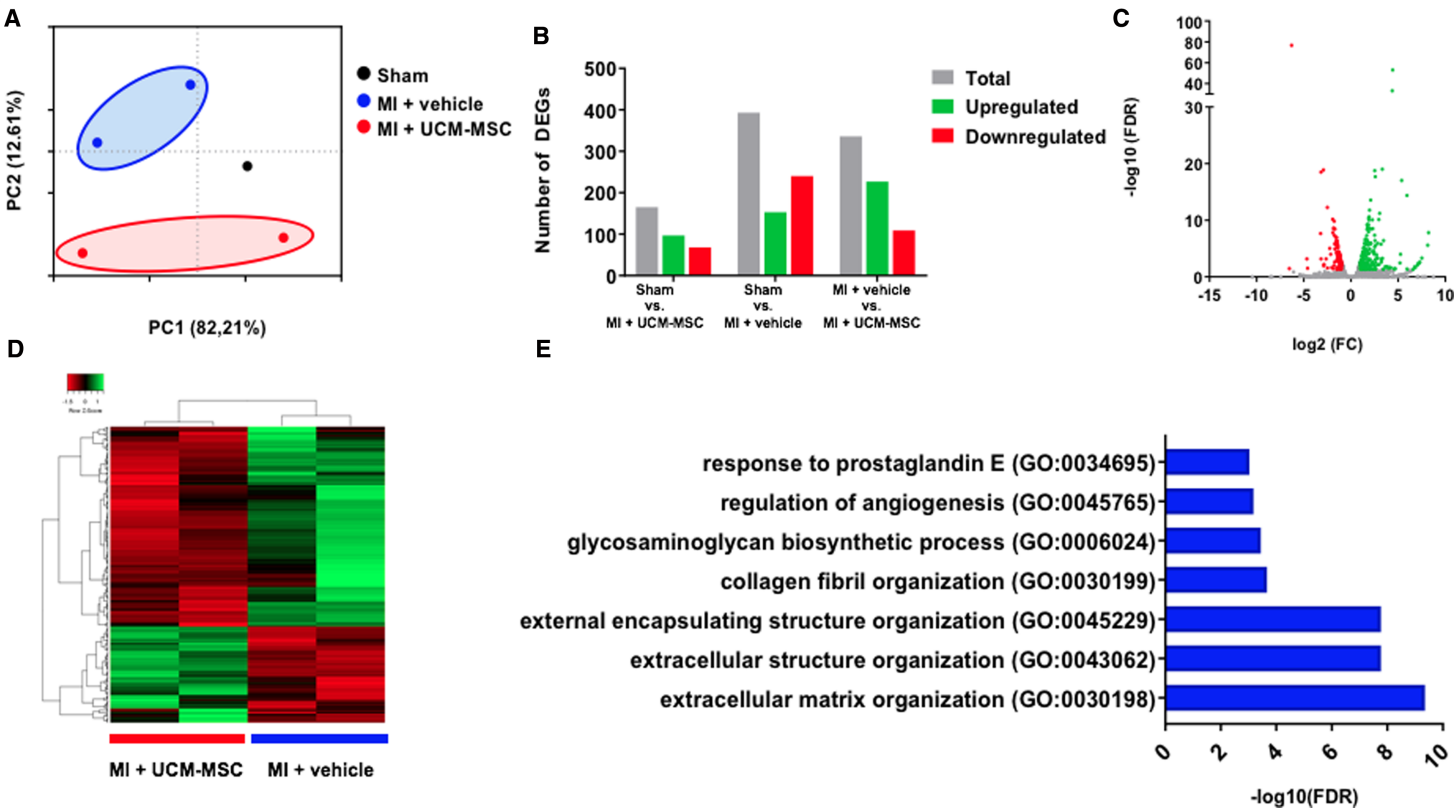
Transcriptional alterations in the remote myocardium after UCM-MSC delivery. (**A**) Principal component analysis (PCA) plot showing the segregation of samples according to gene expression profiles. (**B**) Total number of differentially expressed genes between the 3 groups. (**C**) Volcano plot of MI + Vehicle vs. MI + UCM-MSC comparison with upregulated genes in MI + UCM-MSC colored in red and downregulated genes colored in green. (**D**) Heatmap depicting up and downregulated genes in MI + UCM-MSC. (**E**) Gene ontology enrichment analysis of Biological Processes upregulated in MI + vehicle, organized by false discovery rate (FDR) score.

Differences in gene expression in the remote myocardium were uncovered by RNA-sequencing. AMI-Vehicle and AMI-UCM-MSC-treated animals were segregated in groups in the PC1 axis of the PCA plot (variance of 12.6%) (Figure 7A). Differential gene expression (DEG) analysis (log2FC <±1, FDR < 0.05) unveiled differences in all comparisons tested (Figure 7B). Of note, Sham vs. AMI-hUCM-MSC group yielded the least amount of DEGs, suggesting that the treatment had a positive impact on the molecular program of the infarcted animals. When comparing AMI-vehicle and AMI-hUCM-MSC treated animals, 336 were differentially expressed, as depicted in the volcano plot and heatmap (Figures 7C,D). To unveil which biological processes were altered with the cell therapy, gene ontology (GO) analysis of differentially expressed genes was performed. Regarding upregulated genes in AMI-hUCM-MSC, no significantly regulated processes were detected. Importantly, genes downregulated in AMI-hUCM-MSC treated animals were significantly associated with “extracellular matrix organization”, “glycosaminoglycan biosynthetic process” and “regulation of angiogenesis” (Figure 7E). Upregulated genes in Vehicle include NPPB, COL1A1, FMOD, LOX and MMP9, among others, some of which are related with pro-fibrotic processes and heart failure. These findings are in line with the observed tendency for less interstitial fibrosis found in histological samples from AMI-hUCM-MSC, as compared to AMI-vehicle animals.

### Brain natriuretic peptides and galectine-3

Serum levels of NT-proBNP were similar between groups, ranging from 27.6 to 244.0 pg/ml (Supplementary Figure S4). Serum galectin-3 levels ranged from 0.12 to 6.06 ng/ml. Even though serum levels were numerically lower in the AMI-hUCM-MSC group as compared to AMI-vehicle, differences did not reach statistical significance (Supplementary Figure S5A). Nonetheless, a positive and significant correlation between serum levels of galectine-3 and the burden of interstitial fibrosis in the remote area of the left ventricle was found (Pearson correlation coefficient = 0.54, *p* = 0.032; Supplementary Figure S5B).

## Discussion

To the best of our knowledge, this is the first study to report on the use of an *off-the-shelf* clinical-level GMP-compliant advanced medicinal product of human umbilical cord matrix-derived MSC (hUCM-MSC) in a fully percutaneous pre-clinical model of myocardial infarction with reperfusion, and to thoroughly explore its safety and efficacy in a randomized, sham- and placebo-controlled blinded trial. Our main findings were that intracoronary injection of the cell product, at the selected dose (1) showed no evidence of significant coronary flow impairment; (2) was associated with improved systolic function as evaluated by invasive pressure-volume relationship parameters; and (3) that improvement in mechanical performance did not depend on the reduction of morphological infarct size alone, despite animals treated with hUCM-MSC showed signs of less adverse remodeling.

### Prior studies using hUCM-MSC in experimental myocardial infarction

Most studies of umbilical cord cells in experimental MI have used cord blood-derived hematopoietic and endothelial precursor cells, and other subtypes of stromal cells (28). Available evidence concerning MSC from the cord matrix (Wharton's Jelly) in large-animals is limited to three studies, all of which have used an open-chest LAD ligation model (29–31). Despite significant improvements of infarct size, LV remodeling and ejection fraction have been shown (28), because most patients sustaining an AMI receive reperfusion therapy, permanent coronary ligation models may be poor disease surrogates. We advocate that the closed-chest and reperfusion model that was used in our experiment has several potential translational advantages. Firstly, it better reproduces the setting and pathophysiology of clinical MI. Also, both the percutaneous approach and intra-coronary administration of the cell product are more applicable in clinical practice, especially when envisioning the potential of cell therapy as an *off-the-shelf* product for reperfusion-adjuvant treatment (32). Finally, the less invasive nature of percutaneous approaches improves experimental logistics and decreases risks associated with surgical procedures.

### Safety of intracoronary administration of hUCM-MSC

Prior studies have reported reduced epicardial blood flow (27), microinfarctions and extension of infarct size (33), as well as microvascular disruption (namely assessed by the index of microcirculatory resistance) (34) in relation to intracoronary infusion of MSC. Although these observations warrant concern, putative deleterious effects of IC-injection of MSC are related to a variety of factors, some of which are modifiable. MSC are relatively large cells on their own, and depending on rheologic conditions of the solution they can further aggregate and easily plug the intramyocardial capillary bed. This can be prevented by keeping a low cell concentration (to minimize viscosity) and permanently agitating the solution during injection (as we did). Also, microvascular impairment has been related both to total cell dose (in particular >1 × 10^6^ MSC/Kg of body weight) and infusion rates and, importantly, higher doses of MSC have not been consistently associated with enhanced biological effects (27, 35). With the dose (which was 1.8- to 7.6-fold lower than in studies reporting flow impairment and microvascular disruption) and injection rates used in our experiment we could not detect meaningful effects on coronary flow, as assessed by real time Doppler-derived average peak velocity. However, in animals injected with vehicle solution, an increase in flow velocity occurred throughout the injection (Figure 2). We hypothesize that this could be explained by some degree of hyperaemia induced by the vehicle solution, which may not have been blunted by a minimal residual microvascular effect caused by cells, as APV was in fact numerically higher in placebo than in the hUCM-MSC group at the end of the injection. Still, in cell-treated animals, epicardial flow velocity remained stable and very similar to baseline, suggesting that microvascular disruption (if present) was unremarkable.

### Improvement in LV systolic function

In clinical practice, parameters derived from several imaging modalities—ejection fraction being by far the most widely used—have proved useful for risk stratification and clinical decision making. As such, pre-clinical studies of cell therapy in myocardial infarction models have universally assessed changes in LV function, remodeling and scar size as surrogates of clinical efficacy. We have demonstrated that intracoronary transfer of hUCM-MSC shortly after reperfusion was associated with improved left ventricular systolic function, as assessed by both load-dependent and independent parameters, accurately measured by invasive simultaneous LV pressure and volume recordings. Considering the limited extent of infarct size reduction, the observed improvement in ejection fraction is likely to be also related to other factors such as the positive effects of hUCM-MSC both on LV after-load (as reflected by significantly lower systemic vascular resistance) and intrinsic myocardial contractility, translated into higher Ees*_i_* and PRSW in cell-treated animals, as compared to placebo. In fact, in patients with reperfused acute myocardial infarction, the increase in EF overtime has been shown to be largely and independently linked to a concomitant decline in peripheral vascular resistance, which strengthens the potential clinical implications of our observations (36). However, the mechanisms underlying the beneficial effects on peripheral vascular remodeling are not fully elucidated.

Skinned cardiomyocyte preparations provide a way to assess modifications occurring at the cellular level in both physiological (e.g., stretch) and pathological context (e.g., ischemia) (20). In normal cardiomyocytes the degree of activation of the cardiac myofibrils by calcium rises as muscle length is increased, up to a certain threshold (37). Accordingly, in our specimens, sarcomeres from sham animals exhibited the highest active tension at all lengths. In infarcted animals, for increasing sarcomere lengths, the force of isometric contraction (active tension) was higher in cell-treated animals, as compared to placebo, and closest to the force developed by sham-controls. These findings are in line with invasive hemodynamics and provide a molecular mechanism for the improved load-independent contractile function that we observed in cell-treated animals. Although the exact paracrine mediator has not be identified, MSC have been shown to improve calcium handling and contractile function by interfering with SERCA2a and L-type calcium channel gene expression and modulation of myocyte PI3K/Akt signalling (38).

Because true cardiomyogenic response to cell therapy was not specifically assessed in our experiment, a contribution of newly formed cardiomyocytes to the increased intrinsic contractility cannot be ruled out. In a rat model of reperfused myocardial infarction, it has recently been shown that intramyocardial injection of human umbilical cord-blood derived somatic stromal cells induced a significant thickening of the left ventricular wall with cTnT+/BrdU+ cardiomyocytes, which was mediated by a T-Cell driven regenerative response from endogenous cardiomyocytes (39).

### Infarct size and fibrosis

We found that improvement in myocardial contractility was not matched by a large difference in infarct size as compared to placebo. Although scar extent was in fact smaller in hUCM-MSC treated animals (Figure 4), the difference between groups was not statistically significant. Infarct size (expressed as a percentage of left ventricle area) in placebo animals was within range of prior reports using similar methodology (16). It has been suggested, however, that reporting infarct size as percentages of area or length may be misleading due to relative elongation of the infarcted and non-infarcted ventricle (as a consequence of different degrees of remodeling), and may thus not reflect true changes in actual fibrotic scar (40). Nonetheless, the observed relationship between cell viability and infarct size should be taken into account when interpreting differences in scar extent and, in our view, adds relevance to the *post hoc* analysis performed to address its implications on experimental results. By excluding the one animal treated with a cell product exhibiting a viable cell count much lower than average (76% viable cells; Supplementary Figure S2), the treatment effect of hUCM-MSC on infarct size improved and was statistically significant (Supplementary Figure S3). Also, this highlights the importance of standardization and quality assessment of cell products if a clinical use is envisioned. It has been widely apparent in the literature that the magnitude of biological effects of stem cells is prone to significant variation, even in similar experimental settings (41). This perceived inconsistency is likely to be one of the main reasons undermining translational efforts in the field of stem cell therapy. In the clinical setting of post-MI cardiomyopathy, even though there is a close relationship between such parameters as scar extent and ejection fraction and clinical status, discrepancy may exist between morphological surrogates and residual functional performance of the myocardium. In a metanalysis of 24 randomized controlled trials comparing bone marrow derived cells (BMC) with placebo, the authors found that intracoronary BMC treatment lead to a moderate improvement of LVEF without a clear (statistically) significant effect on infarct size (42).

Different factors and targets may affect the ability of the remaining viable tissue to compensate for the lost contractile mass, the extracellular matrix (ECM) being one of the most important (43). It plays a central role in cardiac homeostasis, acting as a mechanical frame that supports and enhances contraction (44), an active signaling hub that coordinates cell function and interaction and as a reservoir of growth factors and molecules that modulate remodeling and tissue repair (45, 46). The deleterious effects of the expansion of the ECM in the form perivascular and interstitial fibrosis are well recognized. It may jeopardize blood flow and vasodilatory response (47), and interfere with systolic function by compromising excitation-contraction coupling and protease/antiprotease balance by triggering degradation of fibrillar collagens (45). Likewise, many pharmacologic interventions currently recommended to improve outcomes in heart failure with reduced ejection fraction have a favorable impact on non-contractile components of the myocardium, namely inhibition of fibrosis (48). We cannot exclude that treatment with ACE-inhibitors and beta-blockers that was used in our model could have contributed to skew the results concerning scar extent. Still, interstitial fibrosis and cardiomyocyte hypertrophy were further lessened in the remote myocardium of infarcted animals treated with hUC-MSC, as compared to placebo (Figure 4), thus suggesting a favorable effect on these healing mechanisms (49). Consistent with this observation, serum levels of galectine-3—a beta-galactosidase binding protein that has been shown to be associated with cardiac fibrosis and also with mortality in acute and chronic heart failure (50, 51)—correlated well with fibrosis extent and were lower at 8-weeks post-MI in animals treated with hUC-MSC, although the difference was not statistically significant. Likewise, RNA-sequencing showed downregulation of several extracellular matrix organization genes known to exert deleterious effects on ECM synthesis and remodeling after AMI, including MMP-9, TIMP-1 and SERPINE-1 (PAI1), associated with hUCM-MSC treatment (52–55).

Taken together, these observations indicate that a relevant functional effect of cell therapy exists beyond large modifications in macroscopic surrogates (such as infarct size) and that a meaningful interference with injury and repair mechanisms occurs at a molecular and structural level.

### Lack of interference with diastolic function

There were no statistically significant differences in any of the invasive parameters of diastolic function, although numerical estimates favored hUCM-MSC-treated animals. This is in line with the observations concerning interstitial fibrosis in our study. Fibrosis increases myocardial stiffness, which is the hallmark of diastolic dysfunction, but we may hypothesize that the animal model is relatively insensitive to the induction of diastolic dysfunction due to high intrinsic stiffness of the myocardium. This is supported by elevated LV end-diastolic pressures in shams and similar amounts of interstitial fibrosis as compared to infarcted animals treated with placebo. Finally, improvement in systolic function with no difference in diastolic elastance, dP/dT and *tau*—despite significant decreases in interstitial fibrosis—has been reported by others in a similar study of AMI with reperfusion in sheep (27).

### Study strengths and limitations

In addition to randomization, our study included positive and negative controls and, most importantly, all evaluations were performed by blinded operators. Although some of the endpoints did not reach statistical significance—which may also be related to small sample size and type II error—study readouts were consistent with each other and pointed in the same direction. This supports the internal validity of our findings and further minimizes the risk of residual bias. The absence of statistical significance does not exclude a relevant biological effect.

Invasive functional parameters were obtained only at the end of the 8-week observation period. Despite procedural standardization, because animals were not syngeneic and also due to intrinsic biological and unwarranted technical variability, serial (baseline and follow-up) evaluations within each group, would have allowed evaluation of the acute consequences of myocardial infarction, as well as the temporal changes related to cell therapy, which could have strengthened our results.

The limitations of reporting histological infarct size have been previously mentioned. Demonstration of total scar tissue volume or mass is difficult in post-mortem preparations. Advanced imaging modalities such as FDG-Positron Emission Tomography and Cardiac Magnetic resonance with gadolinium late enhancement have excellent correlation with *in vivo* fibrosis and would have provided further insight into morphometric readouts related to the impact of intracoronary hUCM-MSC on infarct extent. Importantly, after the ischemic insult to the myocardium, tissue remodeling and fibrosis occur progressively and depend on many intertwined factors. We cannot exclude that a longer follow-up wouldn't have enhanced differences between AMI groups at the histological level.

Despite including a reperfusion stage, our experimental model lacks the thrombotic component of acute coronary occlusion, which can further worsen with balloon dilatation and stent implantation (56) and influence the response to cell infusion, both in terms of safety and efficacy. Also, because reperfusion is associated with smaller infarcts, the efficacy of intra-coronary treatment in larger and non-reperfused myocardial infarction could not be assessed by our design.

Finally, as we tested only one dose of hUCM-MSC, our findings concerning safety of intra-coronary transfer may not extrapolate to of higher cell numbers or different administration rates.

## Conclusion

Our experiment demonstrated that intracoronary transfer of hUCM-MSC (at the selected dose of 5 × 10^5^ cell/kg) early after reperfusion in a pre-clinical swine model of myocardial infarction was associated with improved left ventricular systolic performance, enhanced cardiomyocyte contractility, favorable modification of myocardial interstitial fibrosis and downregulation of genes related to matrix remodeling. Cell injection was safe in the acute phase. Our findings support further translational investigation of allogeneic hUCM-MSC as adjuvant therapy for acute myocardial infarction.

## Data Availability

The data presented in the study is deposited in ArrayExpress with accession number E-MTAB-13022.
