# Supplementary material for: Human-umbilical cord matrix mesenchymal cells improved left ventricular contractility independently of infarct size in swine myocardial infarction with reperfusion
Source: Front Cardiovasc Med. 2023 Jun 5;10:1186574. doi: 10.3389/fcvm.2023.1186574 (PMC10277821; doi:10.3389/fcvm.2023.1186574)

Human-umbilical cord matrix mesenchymal cells improved contractility independently of infarct size in experimental reperfused myocardial infarction

Luís Raposo[(1),(2),(3)], Rui J. Cerqueira[(4),(5)], Sara Leite[(5),(6),(7)], Liliana Moreira-Costa[(5)],Tiago L. Laundos[(7),(8),(9)], Joana O. Miranda[(5)], Pedro Mendes-Ferreira[(5),(10)], João Almeida Coelho[(5)], Rita N. Gomes[(7),(8),(9)], Perpétua Pinto-do-Ó[(7),(8),(9)], Diana Nascimento[(7),(8),(9)], André P. Lourenço[(5),(11)], Nuno Cardim,[(2,3)]Adelino Leite-Moreira[(4,5)]

(1) Cardiology Department, Hospital de Santa Cruz - Centro Hospitalar de Lisboa Ocidental, Lisbon, Portugal

(2) Hospital da Luz – Lisboa, Luz Saúde, Lisbon, Portugal

(3) Nova Medical School, Lisbon, Portugal

(4) Department of Cardiothoracic Surgery, Hospital Universitário de São João, Porto, Portugal

(5) UnIC@RISE, Department of Surgery and Physiology, Faculty of Medicine of the University of Porto, Porto, Portugal

(6) Anta Family Health Unit, Espinho/Gaia Healthcare Centre, Espinho, Portugal.

(7) ICBAS- Instituto de Ciências Biomédicas Abel Salazar, Universidade do Porto, Porto, Portugal.

(8) I3S – Instituto de Investigação e Inovação em Saúde, Universidade do Porto, Porto, Portugal.

(9) INEB – Instituto Nacional de Engenharia Biomédica, Universidade do Porto, Porto, Portugal.

(10) Paris-Porto Pulmonary Hypertension Collaborative Laboratory (3PH), UMR_S 999, INSERM, Université Paris-Saclay

(11) Department of Anesthesiology, Hospital Universitário de São João, Porto, Portugal

Corresponding author:

Luís Raposo, MD

UNICARV – Hospital de Santa Cruz, CHLO

Av. Prof. Reinaldo dos Santos

1800-067 Carnaxide - Portugal

email: lfor.md@gmail.com

Fax: +351 212421388

Phone: +351 214241380

Supplemental Material

Biological Characterization of the GMP-compliant cell product

The cell product used in the experiment has been widely studied and characterized during the development stage. In brief, flow cytometric analysis, demonstrated that these cells express the surface marker profile defined by the International Society for Cells and Gene Therapy for MSCs (>95% positive for the surface markers CD105, CD90 and CD73 and <2% for CD45, CD34, CD14, CD19 and HLADR). Additionally, CD44, a mediator protein of cell-to-cell interactions, cell adhesion and migration, generally associated with MSC phenotype was also highly expressed. For regulatory purposes the stability of the MSC-GMP-compliant cell product throughout the expansion process has been thoroughly tested. Namely, genetic stability was documented until passage 15, marker expression profile was compliant with ISCT guidelines for at least 20 passages and differentiation potential was maintained to at least P15. For the cell bank, cultures were maintained until passage 7 or 8, yielding a sufficient number of cells within the known safety range. Post-thawed cells maintained the MSC phenotype regarding cell surface markers, genetic stability, differentiation potential, cell morphology, adherence to the plastic surface of the culture flasks and growth behaviour, similar to cells prior to cryopreservation (data not shown). Please refer to references 7 and 17 of the main manuscript for a detailed description.

Baseline Characteristics of study animals

Supplemental Table 1.

Characteristics of study animals at baseline instrumentation.

	Sham (n=8)	AMI+Vehicle (n=12)	AMI+hUCM-MSC (n=11)	p value*
Weight (Kg)	29.1±4.0	31.6±9.7	38.8±8.6	0.258
SBP (mmHg)	111.6±14	113.5±12.3	104.2±10.2	0.302
DPB (mmHg)	68.8±11.7	64.9±14.7	63.4±8.9	0.333
MBP (mmHg)	87.0±13.4	84.4±13.3	79.8±9.8	0.479
CVP (mmHg)	11.0±2.3	10.9±2.4	11.6±3-0	0.614

SBP, Systolic Blood Pressure; DBP, Diastolic Blood Pressure; MBP, Mean Blood Pressure; CVP, Central Venous Pressure; MI, Myocardial Infarction; hUCM-MSC, Human Umbilical Cord Matrix Mesenchymal Stromal Cells
*Analysis of Variance (ANOVA)

Detailed description of the Pressure-Volume relationships at 8-week follow-up

Supplemental Table 2.

Invasive Hemodynamic Parameters obtained 8-weeks after baseline instrumentation.

	Sham + Vehicle (N=6)	AMI + Vehicle (N=7)	AMI + hUCM-MSC (N=6)
Weight (Kg)	37 ± 2	43 ± 3	46 ± 3
BSA (m2)	0.97 ± 0.03	1.04 ± 0.05	1.1 ± 0.04
HR (bpm)	81 ± 8	97 ± 7	92 ± 9
SV*i* (mL/m2)	51 ± 2	33 ± 4*	46 ± 6**
SW*i* (mmHg*L/m2)	4.8 ± 0.5	2.5 ± 0.4*	3.8 ± 0.6**
CI (L/(min*m2))	4.1 ± 0.3	3.1 ± 0.2*	4.1 ± 0.4†
EDV*i* (mL/m2)	63 ± 3	80 ± 5	73 ± 8
ESV*i* (mL/m2)	15.6±3.7	47.4±8.4*	28.5±12.6†
EF (%)	86 ± 3	43 ± 4*	65 ± 6*†
dP/dtmax vs EDV*i* (mmHg*m2/(mL*s))	11 ± 1	9 ± 1	12 ± 2
Pmax (mmHg)	125 ± 10	98 ± 5*	108 ± 6
MaxPowI (mmHg*L*m2/s)	24 ± 7.1	8.4 ± 1.8*	13 ± 3.7
dP/dt max (mmHg/s)	2720 ± 220	1980 ± 220*	2040 ± 60§
Ees*i* (mmHg*m2/mL)	2.9 ± 0.4	2.1 ± 0.7*	2.8 ± 0.4**
V0*i* (mmHg)	79 ± 4	-12 ± 15	7 ± 26
PRSW (mmHg)	99 ± 11	36 ± 4*	75 ± 13*†
SVRi (mmHg/(L/min/m2))	20.3±5.7	26.3±3.1**	18.8±6.24†
Ea*i* (mmHg*m2/mL)	2.3 ± 0.2	3 ± 0.3	2.4 ± 0.3
Tau (ms)	38 ± 2	40 ± 4	35 ± 2
EDP (mmHg)	14 ± 1	13 ± 2	13 ± 2
beta I (m2/mL)	0.17 ± 0.05	0.16 ± 0.05	0.19 ± 0.03
alpha	1.67 ± 1.65	0.17 ± 0.13	0 ± 0
CE (%)	66 ± 4	47 ± 5*	61 ± 7
VVC	1.3 ± 0.2	0.8 ± 0.1§	1.2 ± 0.2

Invasive hemodynamic parameters in sham and infarcted pigs injected with vehicle (Sham and AMI-Vehicle, respectively), and in infarcted animals injected with umbilical cord stromal cells (AMI-hUCM-MSC).

BW, body weight; HR, heart rate; CI, cardiac index; EF, ejection fraction; EDV, end-diastolic volume; ESV, end-systolic volume; EDP, end-diastolic pressure; MAP, mean arterial pressure; τ (Tau), time constant of isovolumic relaxation derived by logistic fitting; E_{es}, end-systolic elastance as derived by linear fitting of the end-systolic pressure volume relationship; PRSW, preload-recruitable stroke work; ß, chamber stiffness constant as derived by exponential fitting of the end-diastolic pressure-volume relationship; SV, Stroke Volume; SW, stroke work; VVC, ventriculo-vascular coupling; SVR, Systemic Vascular Resistance.

*p<0.05 vs. sham-vehicle; †p<0.05 vs AMI-vehicle; § p<0.1 vs. sham-vehicle; ** p<0.1 vs AMI-vehicle

Relationship between hUCM-MSC viability and histological infarct size

Supplemental Figure 1. Cell viability in samples intended for administration in animals randomized for intracoronary hUCM-MSC administration (n=10/11). Viability, measured as percentage of total viable cells per high magnification field (400x) averaged 87±7%, ranging from 76% to 98% (Panel A). Panel B shows a representative example of the trypan blue method for assessment of cell viability; viable MSC, with an intact cell membrane appear white, as opposed to non-viable cells that stain blue.

A

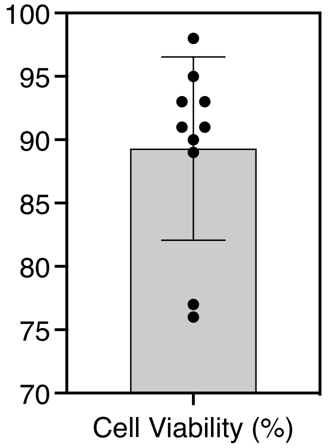

B

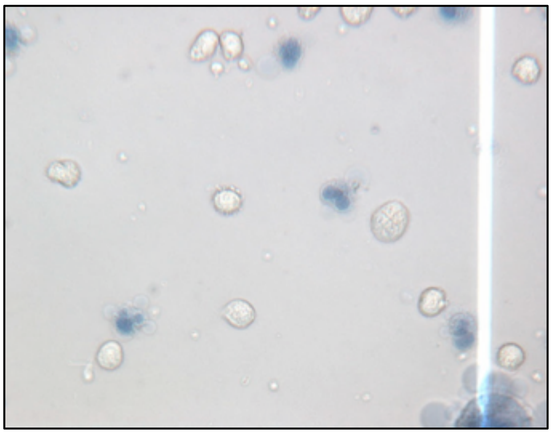

Supplemental Figure 2. Correlation between infarct size and cell viability. Exploratory analysis showed a potential link between the amount of viable cells in the injectate and final infarct size, as measured by midline length and left ventricle area percentage fibrosis. This association highlights the importance of strict quality control and the need for standardization of cell products, given its potential implications in biological effects and experimental outcomes.

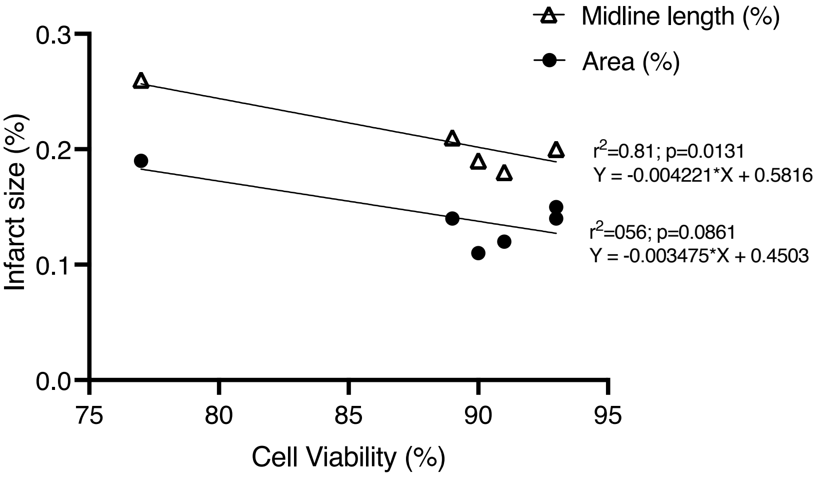

Supplemental Figure 3. Infarct Size as assessed by percent-LV area. Post-hoc analysis of infarct size as assessed by LV area after excluding one animal in which cell viability (76% viable cells) was much lower than average showed a statistically significant decrease in scar area favoring treatment with hUCM-MSC as compared to vehicle.

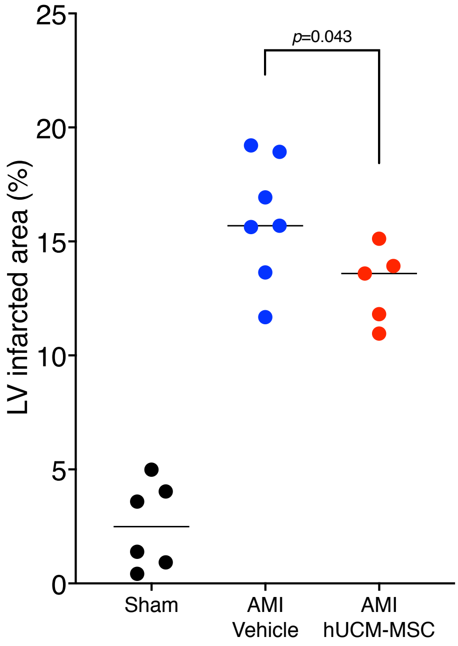

Serum Biomarkers

Supplemental Figure 4. Serum concentrations of NTproBNP **(A)** and Galectine-3 **(B)** in individual animals within each study group. Horizontal lines represent mean and Standard Error (SEM).

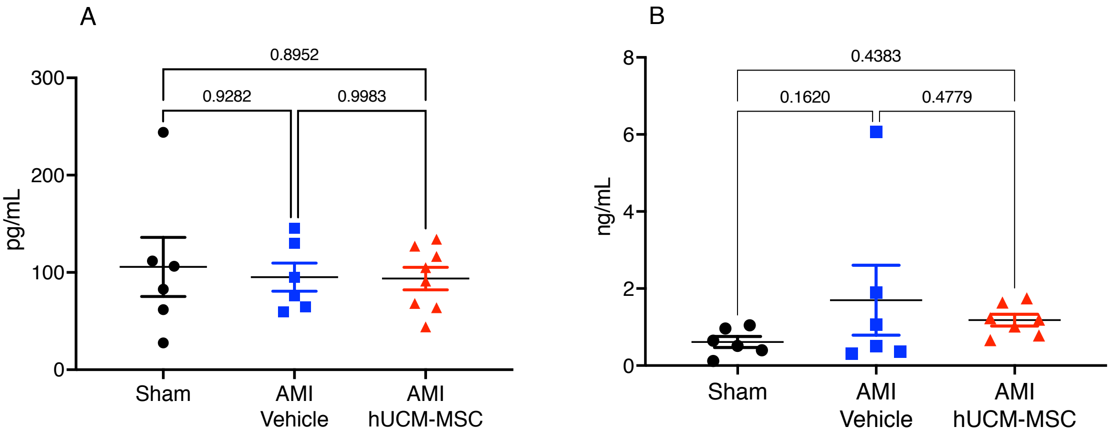

Supplemental Figure 5. Relationship between serum levels of Galectine-3 and interstitial fibrosis. **Panel A** shows the mean and standard error of serum titers of galectine-3 in shams-vehicle (black column), AMI-vehicle (blue) and AMI-hUC-MSC (red) animals. **Panel B** represents the overall positive correlation between the amount of fibrosis deposition in the remote myocardium of the left ventricle and serum levels of galectine-3 (correlation coefficient=0.54 and p=0.032).

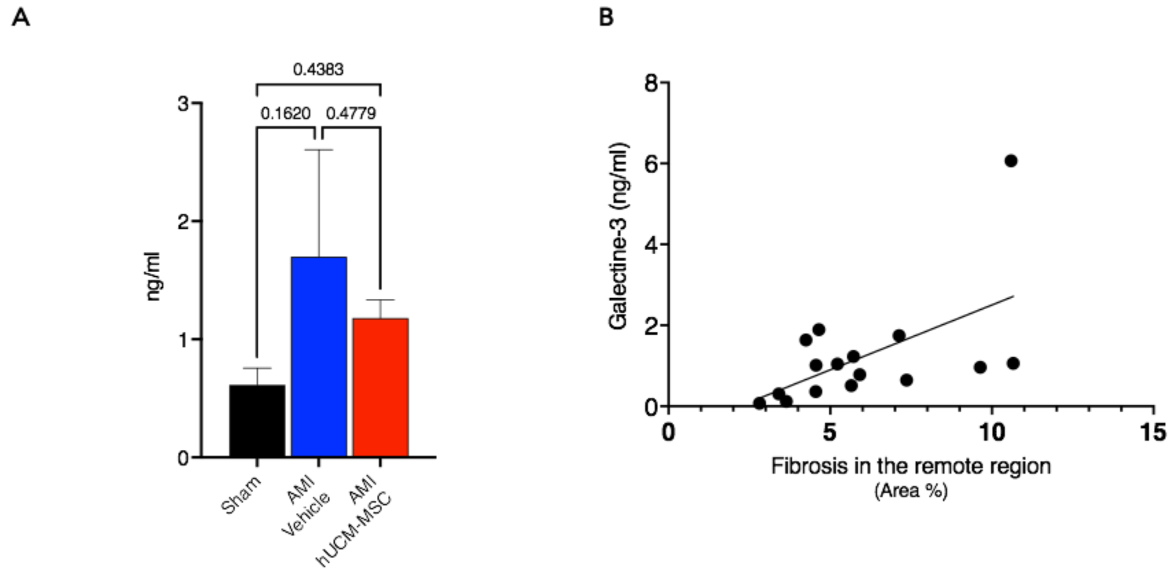

Transthoracic echocardiography

Morphological and functional parameters obtained by transthoracic echocardiography in infarcted animals were intended experimental endpoint evaluation. However, as it was thought to be relevant to the overall interpretation of the study, a summary of available findings is reported.

Although differences did not reach statistical significance, parameters related to LV systolic function, such as ejection fraction, shortening fraction and global longitudinal strain, were all improved in AMI-hUCM-MSC, as compared do AMI-vehicle, thus supporting the findings from invasive measurements. Of note, in AMI animals, estimates of ejection fraction where within range of those obtained invasively.

Supplemental Table 3.

Transthoracic echocardiographic measurements in a sample of 12 infarcted animals with images suitable for analysis at 8-weeks follow-up. Values represent mean and SD within each group.

	EDD (mm)	ESD (mm)	Shortening Fraction (%)	EF(%)	Wall thickness (mm)		GLS(%)	Longitudinal T2P Global
					Mid-Cavity	Apical		
AMI-vehicle (n=5)	38.8±7.3	23.1±7.2	40.0±4.2	43.7±4.1	9.1±1.9	5.4±1.9	-10.2±1.9	374.0±50.5
AMI-MSC (n=7)	35.5±8.5	19.1±8.2	48.6±6.8	58.0±7.1	10.7±2.7	6.7±2.7	-14.3±1.5	407.0±30.2

EF, ejection fraction; EDD, end-diastolic diameter; ESD, end-systolic diameter; GLS, Global Longitudinal Strain; T2P, Time to Peak.

Supplemental Figure 6. Exploratory Echocardiographic parameters of left ventricular systolic function assessed at 8-weeks post-instrumentation according to treatment (vehicle vs hUCM-MSC) in a sample of 12 infarcted animals.

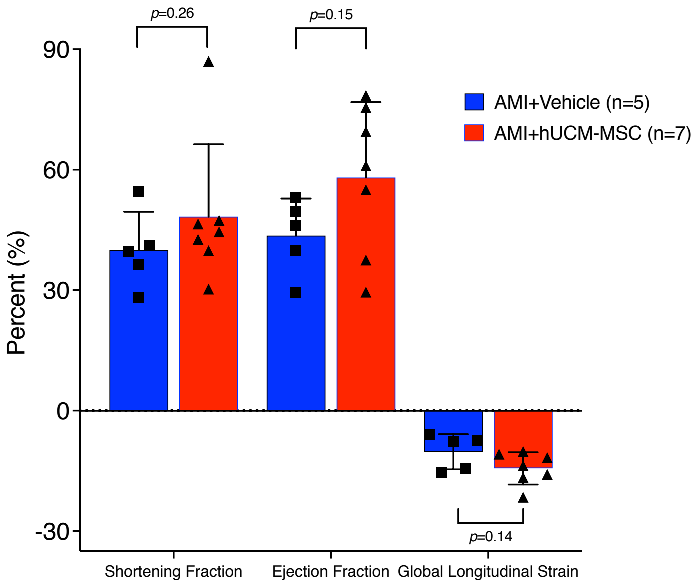

Supplement: Supplementary file 1 [file Datasheet1.pdf]
